# Circulating vascular endothelial growth factor (VEGF) as predictive factor of progression-free survival in patients with advanced chordoma receiving sorafenib: an analysis from a phase II trial of the french sarcoma group (GSF/GETO)

**DOI:** 10.18632/oncotarget.12172

**Published:** 2016-09-21

**Authors:** Loic Lebellec, François Bertucci, Emmanuelle Tresch-Bruneel, Emmanuelle Bompas, Yves Toiron, Luc Camoin, Olivier Mir, Valerie Laurence, Stephanie Clisant, Emilie Decoupigny, Jean-Yves Blay, Anthony Goncalves, Nicolas Penel

**Affiliations:** ^1^ Department of Medical Oncology, Centre Oscar Lambret, Lille, France; ^2^ Department of Medical Oncology, Institut Paoli-Calmette, Marseille, France; ^3^ SIRIC OncoLille, Clinical Research and Methodological Platform, Lille, France; ^4^ Department of Medical Oncology, Centre René Gauducheau, Nantes, France; ^5^ Department of Molecular Pharmacology, Cancer Research Center of Marseille, Institut Paoli Calmettes, Marseille, France; ^6^ Department of Medical Oncology, Gustave Roussy, Villejuif, France; ^7^ Department of Medical Oncology, Institut Curie, Paris, France; ^8^ Clinical Research Unit, Centre Oscar Lambret, Lille, France; ^9^ Department of Medical Oncology, Centre Léon Bérard, Lyon, France

**Keywords:** chordoma, sorafenib, biomarker, placental growth factor, vascular endothelial growth factor

## Abstract

**Background:**

Patients with advanced chordoma are often treated with tyrosine kinase inhibitors without any predictive factor to guide decision. We report herein an ancillary analysis of the the Angionext phase II trial (NCT 00874874).

**Results:**

From May 2011 to January 2014, 26 were sampled. The 9-month PFS rate was 72.9% (95%-CI: 45.9-87.9). During sorafenib treatment, a significant increase in PlGF (18.4 vs 43.8 pg/mL, p<0.001) was noted along with a non-significant increase in VEGF (0.7 vs 1.0 ng/mL, p=0.07). VEGF at D1 >1.04 ng/mL (HR=12.5, 95%-CI: 1.37-114, p=0.025) and VEGF at D7 >1.36 ng/mL (HR=10.7, 95%-CI: 1.16-98, p=0.037) were associated with shorter PFS. The 9-month PFS rate was 92.3% (95%-CI: 56.6-98.9) when VEGF at D1 was ≤1.04 ng/mL versus 23.3% (95%-CI: 1.0-63.2) when >1.04 ng/mL.

**Patients and Methods:**

Chordoma patients were treated with sorafenib 800 mg/day for 9 months, unless earlier occurrence of progression or toxicities. Six biomarkers (sE-Selectin, VEGF, VEGF-C, placental growth factor (PlGF), Thrombospondin, Stem Cell Factor (SCF)) were measured at baseline (day 1: D1) and day 7 (D7).

**Conclusion:**

High levels of VEGF was associated with poor outcome.

## INTRODUCTION

Chordomas are rare primary bone tumors with an incidence lower than 1 case per millions of inhabitants and peak of incidence between 50 and 60 years old [1].

They are derived from undifferentiated notochordal remnants (skull base, mobile spine and sacrum), and the cornerstone of treatment remains surgery with large en-bloc-resection, possible in less than 50% of cases, given the necessary neurological sacrifice and the devastating surgical procedure. Surgery may be followed by high dose radiation therapy (intensity-modulated radiation therapy and stereotaxic therapies, both of which use conventional photons, or hadron therapies) [1–4].

Nevertheless, local and metastatic relapses are frequent, making systemic treatment often discussed. Up to now, there is no standard systemic therapy: conventional chemotherapy is regarded as an inappropriate option [1]; molecularly targeted therapies, particularly imatinib, are often used in first line, despite a low level of evidence based on several phase II trials [5]. As for other very rare cancers, few prospective series testing innovative treatment have been performed

Chordomas express several druggable targets, justifying the use of molecularly targeted therapies, specifically tyrosine kinase inhibitors. Chordomas express stem cell factor receptor (c-KIT), platelet-derived growth factor receptors (PDGFR-α and PDGFR-β), receptor tyrosine-protein kinase erbB-2 (HER2/neu), and epidermal growth factor receptor (EGFR) [1, 6, 7]. High level of VEGF expression has additionally been reported [8, 9]. Sorafenib potently inhibits the proangiogenic vascular endothelial growth factor receptor (VEGFR)-1, VEGFR-2, VEGFR-3, and platelet-derived growth factor receptor-β (PDGFR-β) tyrosine kinases in biochemical assays *in vitro*. In cellular assays, sorafenib inhibits the VEGF-mediated autophosphorylation of VEGFR-2 (human endothelial cells and NIH 3T3 fibroblasts expressing VEGFR-2), and VEGFR-3 [10, 11].

Recently, we conducted a multicenter single-arm phase II trial assessing the activity of sorafenib in unresectable or metastatic chordomas (n=27) [12]. Sorafenib (NSC 724772, BAY 43-9006, Nexavar; Onyx Pharmaceuticals Inc, Everyville, CA; Bayer Healthcare Pharmaceuticals Inc, Wayne, NJ) inhibits VEGFR-1, VEGFR-2, VEGFR-3, and PDGFR-β tyrosine kinases. The best objective response rate was 1/27 (3.7%; 95% CI [0.1-19.0]); the 12-month progression-free rate was 73.0% (95% CI [46.1-88.0]) and the 12-month overall survival rate was 86.5% (95% CI [55.8-96.5]). No predictive factor to identify patients experiencing longer progression-free survival (PFS) has been reported.

In the present ancillary study of this phase II trial, we monitored the circulating level of 6 biomarkers: sE-Selectin (a soluble cell adhesion molecule), VEGF, VEGF-C, thrombospondin, Stem Cell Factor (SFC, ligand of c-Kit receptor), Placental Growth Factor (PlGF: ligand of VEGFR-1). Our aim was to identify predictive factor for longer PFS in such population.

## RESULTS

From May 2011 to January 2014, 27 patients had been enrolled in the ANGIONEXT phase II trial. Among these 27 patients, two blood samples had been taken at D1 and D7 in 26 patients. Clinicopathological characteristics of 26 patients are summarized in Table 1. With a median time of follow-up of 8.7 months (range, 1.3-31.0 months), the 1-year PFS was 72.9% (95% CI 45.9-87.9).

**Table 1:** Clinicopathological characteristics

Characteristics		
Demographic	*N*=26	
Gender		
Male	17	65.4%
Female	9	34.6%
Age		
Median, years [range]	64	[30-86]
**Disease**	*N*=26	
Anatomical location		
Sacrum	20	76.9%
Cervical rachis	3	11.5%
Dorsal rachis	2	7.6%
Lumbar rachis	1	3.8%
Metastatic		
Yes	13	50%
No	13	50%
Number of metastatic sites		
1	5	19.3%
>1	8	30.8%
Main metastatic locations		
Lung	7	26.9%
Bone	5	18.5%
Liver	3	11.5%
Prior treatments	25	96.1%
Surgery	17	65.4%
Radiotherapy	17	65.4%
Chemotherapy or targeted therapy	12	46.2%

**Figure 1:**
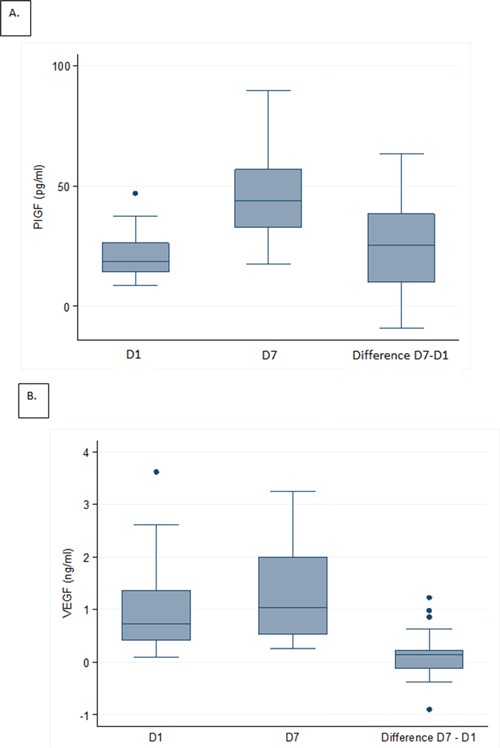
Distributions of serum levels for Placental growth factor (PlGF) and Vascular endothelial growth factor (VEGF) Box plots of serum levels at D1, D7, and difference (D7-D1) for PlGF **A.** and VEGF **B.**

Serum levels of the six tested proteins were measured at D1 and D7. Results are summarized in Table 2. The comparison between values at D1 and D7 showed a significant increase in PlGF circulating level (p<0.001; Figure 1A) and a non-statistically significant increase in VEGF circulating level (p=0.07; Figure 1B). The difference was not significant for the other biomarkers.

**Table 2:** Serum levels at D1 and D7 and difference (D7-D1) between both levels

Variables	Day (D)	Samples *(N)*	Median	Min	Max	Mean	Standard derivation	*p*-value*
VEGF-C (pg/mL)	1	26	4,589.97	865.62	35,235.64	6,618.68	7,507.40	0.38
	7	26	4,882.92	609.50	32,160.37	6,437.91	7,070.01	
	7-1	26	−260.01	−4,669.55	4,906.28	−180.76	1,866.93	
sE-Selectin (ng/mL)	1	26	24.88	12.09	69.77	33.12	16.97	0.62
	7	26	33.02	11.92	62.33	34.11	15.50	
	7-1	26	−0.52	−28.04	21.63	0.99	9.18	
Thrombospondin (ng/mL)	1	26	43,742.79	7,158.35	82,622.85	40,163.85	21,518.19	0.36
	7	26	33,768.88	1,744.26	78,785.90	36,059.75	19,827.65	
	7-1	26	−2,821.24	−52,097.35	47,782.54	−4,104.11	22,627.38	
VEGF (ng/mL)	1	26	0.72	0.09	3.61	1.05	0.87	0.07
	7	26	1.03	0.26	3.24	1.21	0.83	
	7-1	26	0.14	−0.89	1.22	0.16	0.43	
PlGF (pg/mL)	1	26	18.46	8.57	46.89	21.41	9.72	<0.001
	7	26	43.85	17.43	89.75	45.59	17.74	
	7-1	26	25.32	−9.18	63.25	24.18	20.14	
SCF (ng/mL)	1	26	6.38	0.34	11.10	6.68	2.41	0.21
	7	26	7.08	0.44	11.72	6.93	2.62	
	7-1	26	0.09	−1.43	3.03	0.24	0.96	

VEGF: vascular endothelial growth factor; SCF: Stem Cell Factor; PlGF: placental growth factor; sE-Selectin: soluble E-Selectin.

* *p*-value of the comparison between D7 and D1 (D7-D1).

**Figure 2:**
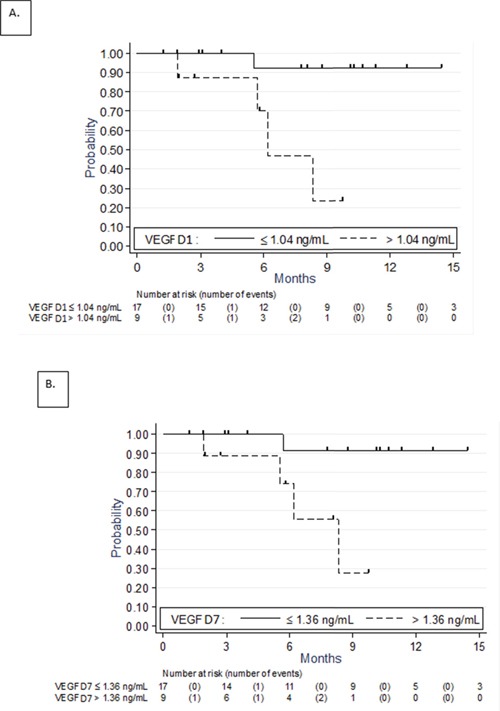
Progression free survival according to VEGF serum levels Kaplan-Meier curves at D1 **A.** and D7 **B.**

The association between PFS and biomarkers levels at D1 and D7 as continuous variable is shown in Table 3. There was no significant association between these parameters and the PFS. However, the association between VEGF values and PFS tended towards significance (p=0.06 at D1 and p=0.08 at D7). The optimal thresholds for maximizing the predictive value of VEGF as binary variable at D1 and at D7 were 1.04 and 1.36 ng/mL, respectively. Such predictive value of VEGF was depicted in Table 4 and Figure 2A and 2B. VEGF at D1 >1.04 ng/mL (HR=12.5, 95%-CI: 1.37-114, p=0.025) and VEGF at D7 >1.36 ng/mL (HR=10.7, 95%-CI: 1.16-98, p=0.037) were associated with shorter PFS. The 9-month PFS was 92.3% (95%-CI: 56.6-98.9) when VEGF at D1 was ≤1.04 ng/mL *versus* 23.3% (95%-CI: 1.0-63.2) when >1.04 ng/mL. The 9-month PFS was 91.7% (95%-CI: 53.9-98.8) when VEGF at D7 was ≤1.36 ng/mL *versus* 27.8% (95%-CI: 1.3-68.4) when >1.36 ng/mL. Given the small number of cases, no multivariate analysis was done.

**Table 3:** Predictive value of biomarkers for progression-free survival (univariate analysis with continuous values)

	HR	95%-CI	*p*
**Value at D1**			
VEGF-C (ng/ml) J0	0.93	(0.70-1.26)	0.66
sE-SELECTIN (ng/ml) J0	0.99	(0.94-1.04)	0.62
THROMBOSPONDIN (μg/ml) J0	0.99	(0.96-1.04)	0.96
VEGF (ng/ml) J0	1.96	(0.98-3.92)	0.06
PIGF (pg/ml) J0	0.98	(0.91-1.06)	0.68
SCF sR/c-kit (ng/ml) J0	0.99	(0.66-1.49)	0.98
**Value at D7**			
VEGF-C (ng/ml) J7	0.91	(0.63-1.31)	0.61
sE-SELECTIN (ng/ml) J7	1.01	(0.96-1.06)	0.70
THROMBOSPONDIN (μg/ml) J7	0.98	(0.93-1.03)	0.39
VEGF (ng/ml) J7	2.15	(0.92-5.06)	0.08
PIGF (pg/ml) J7	1.01	(0.96-1.05)	0.84
SCF sR/c-kit (ng/ml) J7	1.04	(0.74-1.45)	0.83
**Difference (D7-D1)**			
VEGF-C (ng/ml) J7 - J0	0.89	(0.45-1.77)	0.73
sE-SELECTIN (ng/ml) J7 - J0	1.07	(0.99-1.16)	0.08
THROMBOSPONDIN (μg/ml) J7 - J0	0.98	(0.93-1.03)	0.36
VEGF (ng/ml) J7 - J0	0.40	(0.04-3.91)	0.43
PIGF (pg/ml) J7 - J0	1.01	(0.97-1.06)	0.66
SCF sR/c-kit (ng/ml) J7 - J0	1.45	(0.53-3.95)	0.47

**Table 4:** Predictive value for PFS of VEGF at D1 and D7 (univariate analysis with binary values)

	Number of events	6-month PFS rate (IC 95%)	9-month PFS rate (IC 95%)	HR (IC 95%)	*p*-value
**VEGF at D1**					
≤1.04 ng/mL	2/17	92.3% (56.6-98.9)	92.3% (56.6-98.9)	1	
>1.04 ng/mL	4/9	70.0% (22.5-91.8)	23.3% (1.0-63.2)	12.5 (1.37-114)	0.025
**VEGF at D7**					
≤1.36 ng/mL	2/17	91.7% (53.9-98.8)	91.7% (53.9-98.8)	1	
>1.36 ng/mL	4/9	74.1% (28.9-93.0)	27.8% (1.3-68.4)	10.7 (1.16-98)	0.037

**Figure 3:**
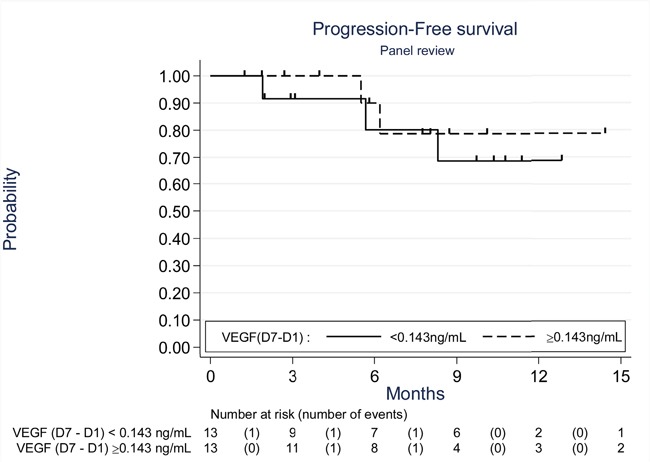
Progression free survival according to the change in VEGF serum levels (D7-D1) (Kaplan-Meier curves) The threshold used (0.143) was the median change in VEGF between D7 and D1.

We compared the groups above and below the median change in VEGF between D7 and D1. There was no significant association between change in VEGF according to the median and the PFS (p=0.87; Figure 3).

## DISCUSSION

In the present study, we hypothesized that sorafenib acts on circulating pro/antiangiogenic biomarkers. To our knowledge, we report here for the first time these changes in chordoma patients receiving an anti-angiogenic treatment. We found that sorafenib significantly increased the serum level of PlGF and tended to increase the level of circulating VEGF. More importantly, we showed that high VEGF levels at D1 and D7 tended to be associated with poor PFS when analyzed as continuous variable and were significantly associated with poor PFS when analyzed as binary variable. Such relationship was particularly strong with high hazard ratios and 95%-CI excluding despite the limited sample size (HR=12.5 [1.37-114.0] for D1 level and HR=10.7 [1.16-98.0] for D7 level). Finally, we showed that the group with greatest rise in VEGF had no more benefit than the group with lowest rise (p=0.87).

Some data are available in other sarcoma subtypes or in more frequent cancer types. Recently, we observed in patients with vascular sarcomas (angiosarcoma and epithelioid hemangioendothelioma) receiving sorafenib an increase in circulating level of VEGF-A [13]. Here also, high level of VEGF-A at baseline was significantly associated with poor PFS: 4.7 months if baseline VEGF-A ≥500 pg/mL vs 34.0 months if <500 pg/mL. Low level of VEGF-A was also significantly associated with best objective response rate, non-progression at 180 days and time to progression [13]. Other published studies have described biomarker changes under sorafenib treatment. In sarcoma patients (whatever the histological subtype) receiving sorafenib, a significant increase of VEGF and PlGF level was also described [14]. In hepatocellular carcinoma patients receiving sorafenib, Llovet et al. noticed that baseline VEGF had prognostic value for overall survival, and trend toward enhanced survival benefit was observed in case of high concentration of SCF at baseline. They have, likely, measured a significant increase in VEGF during treatment [15]. In renal cell cancer patients receiving sorafenib, a shorter overall survival was associated with high baseline VEGF. Level of this biomarker had significantly increased compared with placebo [16]. However, the optimal method for assessing the level of circulating VEGF remains debated [17].

Several other pro/antiangiogenic biomarkers in patients receiving sorafenib have been measured in different studies, such as angiopoietin 2, hepatocyte growth factor, insulin like growth factor 1 or 2, transforming growth factor beta 1, VEGFR-2 or 3, stromal cell derived factor 1 alpha, fibroblast growth factor, epidermal growth factor, carbonic anhydrase IX, tissue inhibitor of metalloproteinase 1 [13–19]. It seems that sorafenib could induce changes in circulating biomarkers. Sorafenib treatment significantly modified the level of circulating PlGF. The PlGF is a member of the vascular endothelial growth factor family. Vascular functions of PlGF remain poorly understood and controversial, but PlGF is known to stimulate endothelial cell growth, migration and survival, to attract angiocompetent macrophages, and determine the metastatic niche. Unlike VEGF, genetic studies have shown that PlGF is specifically involved in pathologic angiogenesis. Therefore, its inhibition would not affect healthy blood vessels, providing an attractive drug candidate [18–20]. Several preclinical trials have been realized. Among them, Richter et al. described *in vitro* for Ewing sarcomas that PlGF suppression provided reduction of metastatic growth by reducing expression of matrix metalloproteinase and invasiveness [21]. Heindryckx et al. assessed inhibition of PlGF in mouse model for hepatocellular carcinoma (HCC). They observed a significantly decrease of tumor burden by inhibiting neovascularization, by decreasing hepatic macrophage recruitment and by normalizing the remaining bloods vessels, thereby decreasing hypoxia and reducing the prometastatic potential of HCC [22]. In a murine model of fibrosarcoma, PlGF showed important effects on vascular remodeling and normalization, altering tumor growth [23]. Then, Kambadakone et al. realized a phase II clinical trial included 20 patients with soft-tissue sarcomas. Patients received neoadjuvant treatment with bevacizumab, followed by bevacizumab and radiation therapy. They observed that median plasma VEGF concentration rose six-fold to seven-fold at 2 weeks after treatment (p<0.0001). Similarly, PlGF concentration increased two-fold throughout neoadjuvant treatment (p<0.0001). However, there was no correlation with the decrease in tumor perfusion parameters [24]. Moreover, PlGF inhibition by sorafenib has shown a potential interest in treatment for age-related macular degeneration. Indeed, Kernt et al. have shown *in vitro* on human retinal glial cells that sorafenib significantly reduced the light-induced overexpression of VEGF-A, PDGF, and PlGF [25, 26].

Sleijfer et al. have found that in non-adipocytic sarcoma treated with pazopanib low circulating VEGFR2 and high level of circulating PlGF at week 12 were associated with several pazopanib-specific toxicities and poorer efficacy [27]. The role of pro-angiogenic factors in conjunctive tissue tumor treated with anti-angiogenic tyrosine kinase inhibitor warrant further clinical investigations.

Our present study displays four limitations. In theory, the present findings required formal validation with an independent prospective chordoma patients. However, this validation is hardly feasible because (i) chordoma is an exceptional cancer (1 case per million of inhabitants), and (ii) sorafenib is not approved for chordoma treatment. However, we think that our findings are of importance since to our knowledge there is no established predictive factor in chordoma patients treated with antiangiogenic agents and because a phase II trial assessing regorafenib is ongoing (NCT02389244). In the next years the cohort of patients treated with regorafenib might become the validation cohort of our study. The second major limitations of our study is the fact that we are not able to separate the prognostic and the predictive value of our findings. Natural history of chordoma is very slow and the gain of PFS described with molecular targeted therapy like sorafenib might be due to the indolent course of disease. Randomization is required to clearly identify the drug activity (predictive factor) and the natural history of the disease (prognostic) [5, 28]. The current phase II trial assessing regorafenib and conducted by the French Sarcoma Group is a randomized phase II trial placebo versus regorafenib (NCT02389244). The internal comparator, the patients exposed to regorafenib, will be of major importance to identify prognostic factors and analysis the interaction between drug activity and natural course of the disease. Our group has to complete the accrual before addressing the issue of what is predictive and what is prognostic. Moreover, the choice of measured biomarkers is arbitrary in this exploratory study; one could suggest other putative biomarkers [13]. Lastly, the sample size was small thus limiting the statistical power of our results. We did not explore the potential thresholds of other circulating biomarkers to avoid Alpha inflation caused by multiple statistical tests. Regardless its limitations, the present ancillary analysis is the first study identifying chordoma patients benefiting from anti-angiogenic agent.

In France, a new clinical trial assessing the activity/safety of anti-angiogenic tyrosine kinase inhibitor is ongoing; this new trial is a randomized placebo-controlled phase II trial with regorafenib.

In conclusion, our results suggest that VEGF monitoring could be useful for identifying responding chordoma patients under sorafenib. A confirmatory study on a larger cohort is needed, but hardly feasible. We have to better understand the role of the VEGF and PlGF in human chordoma.

## MATERIALS AND METHODS

### Clinical trial

The efficacy and toxicity results of the phase II were previously reported [12]. The following key eligibility criteria were histologically proven metastatic or locally advanced chordoma not amenable to radiotherapy or curative-intent surgery after multidisciplinary decision-making. Prior systemic treatment for chordoma was allowed. The treatment consisted of sorafenib at 400 mg per oral twice daily. Study investigations were conducted after approval by the regional Ethics Committee (“Comité de Protection des Patients Nord-Ouest III”, date of approval: June 16, 2009) and after declaration to the French Health Products Safety Agency (“Agence Française de Sécurité Sanitaire et des Produits de Santé”, date of approval: 1 June 2009). Informed consent was obtained from each patient. This study was registered in the European Clinical Trials Registry (EudraCT N° 2007-004651-10) and ClinicalTrial.gov site (Number: NCT 00874874). The study was conducted in agreement with the Declaration of Helsinki and the International Conference on Harmonization of Good Clinical Practice guidelines. http://www.cancer.gov/clinicaltrials/search/view?cdrid=633547&version=healthprofessional)

### Biomarkers analysis

Twenty-seven patients had been enrolled in this study, but samples were not done in one case: the present cohort thus included 26 patients. Serum samples were collected at baseline (Day 1: D1) and at day 7 (D7), in 5 milliliters Serum-Separating tubes (SST). Blood was centrifuged at 3 000 rounds per minute for 15 minutes. Plasma was transferred to 2 cryotubes labeled and frozen, as soon as possible, at – 80 degrees C, in each center. Those tubes were, then, carried, in containers filled with dry ice to observe cold chain integrity, to anti-tumour Pharmacology Laboratory, in Oscar Lambret Center, Lille. ELISA methods were used as previously described [13] to measure circulating biomarkers: VEGF-C in picograms per milliliter by Quantikine human VEGF Immunoassay (R&D, Minneapolis, USA), sE-Selectin in nanograms per milliliter by Human sE-Selectine Immunoassay (R&D, Minneapolis, USA), thrombospondin in nanograms per milliliter by Human TSP-1 immunoassay (Neogen, Lexington USA via interchim), VEGF in nanograms per milliliter by Quantikine human VEGF Immunoassay (R&D, Minneapolis, USA), SCF in nanograms per milliliter by Quantikine human SCF Immunoassay (R&D, Minneapolis, USA), and PlGF in picograms per milliliter by Quantikine Human PlGF Immunoassay (R&D, Minneapolis, USA). Each sample was analyzed in duplicate and the average value was used for correlations with clinical outcome.

### Statistical analysis

Patient's characteristics were described using percentages and frequencies in case of categorical data and median and extreme values or means and standard deviation in case of continuous data. Biomarkers values at D1 and D7 were compared using paired Student t test or Wilcoxon rank test. Our clinical endpoint was the PFS defined as the time between inclusion and the date of progression according to RECIST 1.1. The response to treatment was assessed according to the RECIST 1.1 guidelines by comparing unidimensional tumor measurements (computed tomography scans) in pre- and per-treatment imaging studies at 2, 4, 6 and 9 months. An independent third party radiologist reviewed selected imaging studies carried out during the treatment period with the study drug to ensure the consistent and unbiased application of RECIST. The predictive value of biomarkers (as continuous variable) for PFS was explored using univariate Cox model. Survivals were calculated using the Kaplan-Meier method and curves were compared with the log-rank test. The level of significance was set up at 0.05. The computer software used for statistical analyses was Stata 13.1 (StataCorp. 2013. Stata Statistical Software: Release 13. College Station, Tx: StataCorp LP).
